# Patient Understanding of Chemotherapy and Goals of Care as Provided by Different Care Team Members

**DOI:** 10.1007/s13187-022-02251-y

**Published:** 2023-01-03

**Authors:** Shanzay Suhail, Sandeep Basu, Sameer A. Batoo, Eyad S. Al-Hattab, Ola Kanj Ahmed, Barbara J. Eidahl, Scott H. Okuno

**Affiliations:** 1 University of WI – Eau Claire, Eau Claire, WI USA; 2 Oncology/Hematology, Mayo Clinic Health System – Northwest Wisconsin region, 1400 Bellinger St, Eau Claire, WI 54702 USA; 3 Nursing Administration, Mayo Clinic Health System – Northwest Wisconsin region, Eau Claire, WI USA

**Keywords:** Cancer, Education, Learning

## Abstract

Chemotherapy can be challenging and overwhelming for patients, but when patients are knowledgeable about chemotherapy, their comfort level, overall satisfaction, and coping improve. It is currently unknown whether patients prefer information about chemotherapy to be provided by specific care team members and whether demographic characteristics affect learning preferences. We developed a 31-question questionnaire that asked patients when chemotherapy information was discussed and who they wanted that information to come from. The questionnaire was given to 50 patients who had completed 1 cycle of chemotherapy. Patients were evenly distributed among age categories of 45 to 64 years, 65 to 74 years, and 75 years or older. Thirty participants (60%) were women, 33 (66%) had high school degrees, and 23 (46%) were receiving their first chemotherapy regimen. Sixty percent of patients best understood goals of care from oncologists, 70% wanted goals of care to come from oncologists, and 61% best understood and wanted to understand logistics of chemotherapy from oncologists. Sixty-six percent of patients understood adverse effects when they were explained by nursing staff, and 56% wanted explanations of adverse effects to come from nursing staff. Patients did not prefer a specific care team member or information source when receiving financial cost information. Patients often preferred to receive chemotherapy information from their oncologist; however, other members of the care team also provided information to patients in a way that was understood.

**Supplementary Information:**

The online version contains supplementary material available at 10.1007/s13187-022-02251-y.

## Introduction

Understanding the many aspects associated with chemotherapy can be both challenging and overwhelming for a patient [1]. However, when patients can anticipate adverse effects and are engaged while learning about the chemotherapy process, their distress is reduced [2]. Furthermore, patients with better chemotherapy understanding are more satisfied and cope better [3]. As care teams become increasingly prevalent, the role of care team members continues to evolve. Because patients are more commonly presenting with numerous comorbid conditions, a team-based approach that includes allied health professionals, social workers, and nurses, alongside physicians, benefits the patient [4]. Care teams may also improve support for patients by exploring their needs and helping them receive information from various domains across the health care system.

However, it is not known whether patients better understand information received from different members of the care team, if patients have a preference about receiving chemotherapy information from specific care team members, or if differences in patients’ understanding and learning preferences vary with demographic characteristics such as sex, education level, age, or the number of prior chemotherapy regimens. To help care team members most effectively allocate their time to meet patient needs, the current study aimed to better understand who provided the most beneficial information about various aspects of chemotherapy to patients, as well as the best time to provide this information. To assess patients’ understanding and learning preferences about the chemotherapy information delivered by oncology care team members, we developed and administered a 31-question questionnaire that was filled out by patients receiving chemotherapy.

## Methods

This study was approved by the Mayo Clinic Institutional Review Board. Oral consent was obtained from study participants. By completing the questionnaire, patients indicated their consent to participate in the study. They did not sign a separate consent form.

We developed an 11-item questionnaire, with a total of 31 questions that collected demographic information (age range, sex, education level, number of chemotherapy regimens, and primary tumor site) and asked questions pertaining to 4 domains of chemotherapy: goals of care, adverse effects, financial costs, and logistics of treatment (Appendix). For each domain, we asked who provided information to the patient, who the patient best understood, and who the patient wanted to best understand. We also asked about when the information was provided and when the patient wanted to receive information. For questions about information sources, possible answers were as follows: oncologist, chemotherapy nurse (nurse administering chemotherapy and monitoring patients), oncology nurse (nurse working in the oncology department), chemotherapy class (group class in which adverse effects and other relevant information were discussed), pharmacist, pamphlets or other documents provided, cancer guide (social worker within the oncology department), and websites or other resources. For questions about when information was or could be provided, “right before beginning treatment” was defined as the moment the patient was preparing for the day’s treatment in the chemotherapy unit. The questions were reviewed by study staff and medical personnel for clarity.

The questionnaire was offered to chemotherapy patients receiving care at Mayo Clinic Health System (Eau Claire, Wisconsin) in the spring of 2019. Patients were asked to complete the questionnaire when they were starting cycle 2 of their first-, second-, or third-line chemotherapy regimen. Questionnaires were given to patients before the second cycle of chemotherapy began; questionnaires were offered until 50 completed surveys were returned. Patients received the questionnaire from rooming staff, i.e., the individuals within the oncology department who led the patient from the waiting room to the examination room (not part of the patient’s care team); completed questionnaires were returned to the front desk as the patient left the cancer center. Patients were not required to complete the questionnaire, and no incentives were offered for survey completion.

### Data Analysis

Data were collated, stratified by demographic characteristics, and analyzed with descriptive statistics. The data from “chemotherapy nurses” and “oncology nurses” were pooled to form the combined response category of “nursing staff” because patients often selected both nursing groups in their response. Frequency and percentage values of responses to each question were calculated for the overall cohort and after stratifying respondents by demographic characteristics. Demographic-specific findings are shown only when results from subgroups differed from those of the overall cohort. We used this analytic approach because it allowed comparisons of the preferences of certain demographic groups to the overall patient population served at the clinic. Free-text comments were summarized, such as comments qualifying the highest educational level (i.e., associate’s degree or some college but no bachelor’s degree).

## Results

Patients were offered questionnaires until we had collected 50 completed questionnaires. Patient characteristics are described in Table 1. Thirty patients (60%) were female, 23 (46%) were receiving their first-line chemotherapy regimen, and 33 (66%) had a high school degree. Of the individuals with a high school degree, 12% listed having some education beyond high school (i.e., associate’s degree, technical degree, or some college). Primary tumor sites were not mutually exclusive (some patients indicated multiple primary tumor sites). Table 2 shows data for the overall study population and for demographic subgroups (whenever subgroup preferences differed from those of the overall group). Whenever subgroups expressed equal preference for 2 different care team members or times, both preferences were described, regardless of whether 1 preference matched the overall study population preference.

**Table 1:** Demographic characteristics and primary tumor sites (*N* = 50)

Characteristic	No. (%)
Age, y	
25–44	2 (4)
45–64	16 (32)
65–74	17 (34)
≥ 75	15 (30)
Sex	
Female	30 (60)
Male	19 (38)
Not answered	1 (2)
Highest education level attained	
High school^a^	33 (66)
Bachelor’s degree	11 (22)
Graduate degree	6 (12)
Chemotherapy regimen	
First	23 (46)
Second	18 (36)
Third	8 (16)
Not answered	4 (8)
Primary tumor site^b^	
Bone marrow	4 (8)
Bones and joints	3 (6)
Brain and nervous system	1 (2)
Breast	11 (22)
Digestive system	3 (6)
Kidney, bladder, and ureter	2 (4)
Lungs	7 (14)
Lymph nodes	7 (14)
Melanoma	2 (4)
Prostate	2 (4)
Skin	2 (4)
Soft tissue	1 (2)
Uterus and cervix	3 (6)
Other	8 (16)

^a^Of the patients with a high school degree, 6 (12%) had education beyond high school (i.e., associate’s degree, technical degree, or some college)

^b^Percentages sum to more than 100% because some patients selected more than 1 primary site

**Table 2:** Patient understanding of chemotherapy goals of care, adverse effects, financial costs, and logistics

Learning preference	Goals of care		Adverse effects		Financial costs		Logistics	
	Overall	Subgroup	Overall	Subgroup	Overall	Subgroup	Overall	Subgroup
Who did the patients best understand?	Oncologist, 60%	Men Oncologist, 56% Nursing staff, 56% Second regimen Oncologist, 60% Nursing staff, 60%	Nursing staff, 66%	First regimen Oncologist, 59% Second regimen Oncologist, 60% Nursing staff, 60% Third regimen Oncologist, 88% Age 25–44 y Oncologist, 100%	Website or other resource, 13%	Men Nursing staff, 30% Website or other resource, 30% Women Pharmacists, 25% Pamphlets, 25% Website or other resource, 25% First regimen Pamphlets, 38% Second regimen Nursing staff, 29% Pharmacist, 29% Website or other resource, 29% Third regimen Pharmacist, 50% Age 45–64 y Pamphlets, 40% Age 65–74 y Nursing staff, 22% Pharmacist, 22% Pamphlets, 22% Website or other resource, 22% Age ≥ 75 y Nursing staff, 25% Pharmacist, 25% Website or other resource, 25% Bachelor’s degree Nursing staff, 50% Graduate degree Pharmacist, 66%	Oncologist, 61%	Men Oncologist, 56% Nursing staff, 56% First regimen Nursing staff, 57% Age 25–44 y Oncologist, 50% Chemotherapy class, 50% Graduate degree Oncologist, 67% Nursing staff, 67%
Who did patients want information from?	Oncologist, 70%	NA	Nursing staff, 56%	First regimen Oncologist, 80% Second regimen Oncologist, 71% Third regimen Oncologist, 100% Age 25–44 y Oncologist, 100% Age 65–74 y Oncologist, 56% Nursing staff, 56%	Oncologist, 13%	Men Oncologist, 18% Nursing staff, 18% Pamphlets, 18% Website or other resource, 18% Women Oncologist, 31% Chemotherapy class, 31% First regimen Chemotherapy class, 40% Second regimen Nursing staff, 29% Pharmacist, 29% Third regimen Oncologist, 33% Pharmacist, 33% Age 45–64 y Nursing staff, 38% Age 65–74 y Nursing staff, 30% Age ≥ 75 y Oncologist, 29% Website or other resource, 29% High school degree Oncologist, 24% Website or other resource, 24% Bachelor’s degree Nursing staff, 40% Graduate degree Oncologist, 33% Pharmacist, 33% Website or other resource, 33%	Oncologist, 61%	Age 25–44 y Oncologist, 50% Chemotherapy class, 50% Age 45–64 y Nursing staff, 67% Graduate degree Nursing staff, 80%
When was information provided?	During oncologist visit, 52%	Men During oncologist visit, 56% Immediately before treatment began, 56% Age 25–44 y Immediately after diagnosis, 50% During oncologist visit, 50% Age 65–74 y Immediately before treatment began, 59%	During oncologist visit, 50%	Women Immediately before treatment began, 48% Second regimen Immediately before treatment began, 53% Age 25–44 y Immediately after diagnosis, 50% During oncologist visit, 50% Age 45–64 y Immediately before treatment began, 57% Age 65–74 y During oncologist visit, 59% Immediately before treatment began, 59% Graduate degree During oncologist visit, 50% Immediately before treatment began, 50%	During oncologist visit, 20%	First regimen Immediately before treatment began, 67% Age 65–74 y Immediately after diagnosis, 43% During oncologist visit, 43% High school degree During oncologist visit, 40% Immediately before treatment began, 40%	During oncologist visit, 61%	Age 25–44 y Immediately after diagnosis, 50% Immediately before treatment began, 50% Bachelor’s degree During oncologist visit, 45% Immediately before treatment began, 45%
When did patients want information provided?	During oncologist visit, 52%	Second regimen Immediately before treatment began, 47% Age 25–44 y Immediately after diagnosis, 50% During oncologist visit, 50% Age 65–74 y During oncologist visit, 53% Immediately before treatment began, 53%	During oncologist visit, 48%	Women Immediately before treatment began, 52% Second regimen Immediately before treatment began, 47% Age 25–44 y Immediately after diagnosis, 50% During oncologist visit, 50% Age 65–74 y Immediately before treatment began, 65% Age ≥ 75 y Immediately after diagnosis, 38% During oncologist visit, 38%	During oncologist visit, 30%	Second regimen Immediately after diagnosis, 50% During oncologist visit, 50% Age ≥ 75 y Immediately after diagnosis, 33% During oncologist visit, 33%	During oncologist visit, 55%	Age 25–44 y Immediately after diagnosis, 50% Immediately before treatment began, 50%

*NA*, not applicable

### Goals of Care

Responses to the questions about the goals of care are summarized in Table 2. Patients wanted information about the goals of care to come from the oncologist and understood them best when they were described by the oncologist. They wanted and received information about the goals of care during their oncologist visits.

### Adverse Effects of Chemotherapy

Responses to the questions about the adverse effects of chemotherapy are summarized in Table 2. Patients wanted to receive and best understood information about adverse effects from nursing staff (chemotherapy and oncology). They also wanted and received information about adverse effects during their oncologist visits.

### Financial Cost of Chemotherapy

Responses to the questions about the financial cost of chemotherapy are summarized in Table 2. In the overall cohort, patients indicated that they best understood information about financial costs when it was provided by websites or other resources. As a group, patients thought they would best understand cost information from the oncologist, but the analysis showed that no demographic subgroup thought that the oncologist could help them the most. Generally, patients received financial cost information during the oncologist visit.

### Logistics of Chemotherapy

Responses to the questions about the logistics of chemotherapy are summarized in Table 2. We defined logistics of chemotherapy as the scheduling and sequencing of treatment. Overall, patients wanted this information from the oncologist, received this information from the oncologist, and best understood the oncologist. Likewise, they wanted and received this information during their oncologist visits. Trends in preferences were not consistent when responses were stratified by demographic characteristics.

## Discussion

Chemotherapy education can greatly affect the patient’s treatment experience, so it is important to understand which members of the care team are most beneficial for the patient’s understanding of various aspects of chemotherapy, including goals of care, adverse effects, financial costs, and logistics, as well as determining what patients believe is the most appropriate time to provide this information. Numerous studies have shown that when patients have a thorough understanding of the goals of care, they are able to make more informed decisions that are aligned with their personal goals, which in turn affects their overall experience [5–9].

With our questionnaire, we learned that patients wanted to receive information regarding various aspects of chemotherapy from the oncologist, although patients also wanted other members of the care team to help them best understand certain aspects of chemotherapy. Patients wanted to discuss the goals of care with the oncologist, the adverse effects with the oncologist and nurses, the financial costs with multiple care team members, and the logistics with the oncologist. Patients stated that information was provided to them during their oncologist visits and that they wanted the information provided at that time. Patients valued the role various care team members had in providing chemotherapy information, as evidenced by the areas where different members of the care team excelled. However, patient preferences from this study suggest that the oncologist should continue to have the greatest role in providing patient education.

From these findings, we recommend that the oncologist should continue to provide the most information regarding the goals of care because they are the care team member that patients best wanted to understand. Furthermore, the oncologist should modify how they provide information about adverse effects to the patient. Possible changes could include providing information by using different language, dedicating more time to the conversation, or a combination of various changes. Future studies could explore the changes that patients would like to see before specific change recommendations are made. Finally, the cancer guide, chemotherapy class, and documents provided to the patient should include information about the financial cost of chemotherapy. The study results also suggested that patients would like the oncologist to provide information in other ways or spend more time with patients to go over financial cost information, but again, further studies are required to determine the specific changes in the care team that patients would like.

We acknowledge some limitations to our current results. For example, the sample size of 50 patients may not be truly representative of the patient population at the clinic. We do not know how many questionnaires were distributed in the clinic before 50 patients returned a completed questionnaire (response rate could not be calculated). Also, we primarily focused on understanding the opinions of patients, without taking into consideration the specific changes that they would appreciate from the care team. Patients seemed to want and expect the oncologist to be responsible for most of the patient education; however, this approach may not be feasible in a real-world cancer care setting, and we did not evaluate possible changes that could meet both patient expectations and provider roles.

Future studies should be conducted to determine why patients best understood adverse effects from nurses, why the oncologist did not meet their expectations regarding what they expected the oncologist to provide, what adverse effect information patients wanted from oncologists compared with from nurses, if there are differences in how care team members provide information to patients that is more or less beneficial for their understanding, and why the oncologist and nurses had limited benefit in the patient’s understanding of financial costs, even though they provided information. Future studies must also consider alternative suggestions for patient education methods when expanding the oncologist’s role is not feasible. Our recommendations are summarized in Table 3.

**Table 3:** Summary of findings and recommended changes

Chemotherapy domain	Who provided information	Who patients best understood	Who patients wanted to best understand	Recommendation
Goals of care	Oncologist and nurses	Oncologist	Oncologist	Oncologist should continue to provide most information
Adverse effects	Oncologist and nurses	Nurses	Oncologist and nurses	Oncologist should modify how information is presented^a^
Financial costs	Oncologist, nurses, and pharmacist	Pamphlets, pharmacist, and websites or other resources	Oncologist, cancer guide, chemotherapy class, pharmacist, and websites or other resources	Cancer guide, chemotherapy class, and websites or other resources should provide information Oncologist should modify how information is presented^a^
Logistics	Oncologist	Oncologist	Oncologist	Oncologist should continue to provide most information

^a^Modifications could include providing information using different language, dedicating more time to the conversation, or implementing a combination of various changes

As care teams become more common in medical practice, it is crucial that we know how to meet patients where they are and to assign tasks to the appropriate care team member to best serve the patient.

## Supplementary Information

Below is the link to the electronic supplementary material.

Supplementary file1 (DOCX 31 KB)

## Data Availability

All relevant, de-identified data supporting the findings of this study are reported within the article and the Appendix.
