# Supplementary material for: Patient Understanding of Chemotherapy and Goals of Care as Provided by Different Care Team Members
Source: J Cancer Educ. 2023 Jan 3;38(4):1215–23. doi: 10.1007/s13187-022-02251-y (PMC10366317; doi:10.1007/s13187-022-02251-y)
Supplement: Supplementary file 1 — Supplementary file1 (DOCX 31 KB) [file 13187_2022_2251_MOESM1_ESM.docx]

**Appendix. Study Questionnaire.**

**The goal of this questionnaire is to determine how patients understand information about chemotherapy from different care team members. By filling out this questionnaire, you are consenting to be a part of this study.**

1. Sex

| 🞎 Male | 🞎 Female |
| --- | --- |

1. Age Group

| 🞎 ≤24 years | 🞎 25-44 years | 🞎 45-64 years | 🞎 65-74 years | 🞎 75+ years |
| --- | --- | --- | --- | --- |

1. Education Level

| 🞎 High school education | 🞎 Bachelor’s degree | 🞎 Graduate degree |
| --- | --- | --- |

1. Primary Tumor Sites (Where did the Tumor Start?)

| 🞎 Digestive system | 🞎 Lung | 🞎 Bones & joints | 🞎 Soft tissue |
| --- | --- | --- | --- |
| 🞎 Skin | 🞎 Breast | 🞎 Brain & nervous system | 🞎 Kidney, bladder, ureter |

🞎 Lymph node 🞎 Bone Marrow 🞎 Head and Neck 🞎 Prostate

🞎 Uterus, cervix 🞎 Melanoma 🞎 Other

1. What chemotherapy/systemic therapy regimen (drug or combination of drugs) are you receiving? (i.e. if you have changed drugs or combinations of drugs once, you would select second)

| 🞎 First | 🞎 Second | 🞎 Third or more |
| --- | --- | --- |

1. A. Did you receive information about the goals of chemotherapy?

| 🞎 Yes | 🞎 No |
| --- | --- |

B. If yes, who provided this information? (check all that apply)

| 🞎 Oncologist | 🞎 Oncology nurse | 🞎 Chemotherapy nurse |
| --- | --- | --- |
| 🞎 Chemotherapy class | 🞎 Pharmacist | 🞎 Pamphlets/documents provided |
| 🞎 Cancer Guide | 🞎 Website/Other ______________________________ | |

C. Who helped you understand the goals of chemotherapy the best? (check ONE)

| 🞎 Oncologist | 🞎 Oncology nurse | 🞎 Chemotherapy nurse |
| --- | --- | --- |
| 🞎 Chemotherapy class | 🞎 Pharmacist | 🞎 Pamphlets/documents provided |
| 🞎 Cancer Guide | 🞎 Website/Other ______________________________ | |

D. Who did you most want to help you understand the goals of chemotherapy? (check ONE)

| 🞎 Oncologist | 🞎 Oncology nurse | 🞎 Chemotherapy nurse |
| --- | --- | --- |
| 🞎 Chemotherapy class | 🞎 Pharmacist | 🞎 Pamphlets/documents provided |
| 🞎 Cancer Guide | 🞎 Website/Other ______________________________ | |

E. When was this information provided?

| 🞎 Immediately following diagnosis | 🞎 During your oncologist visit | 🞎 Right before beginning treatment |
| --- | --- | --- |
| 🞎 Other ______________________________________________________________ | | |

F. When did you want this information provided?

| 🞎 Immediately following diagnosis | 🞎 During your oncologist visit | 🞎 Right before beginning treatment |
| --- | --- | --- |
| 🞎 Other ______________________________________________________________ | | |

1. A. Did you receive information about the side effects of chemotherapy?

| 🞎 Yes | 🞎 No |
| --- | --- |

B. If yes, who provided this information? (check all that apply)

| 🞎 Oncologist | 🞎 Oncology nurse | 🞎 Chemotherapy nurse |
| --- | --- | --- |
| 🞎 Chemotherapy class | 🞎 Pharmacist | 🞎 Pamphlets/documents provided |
| 🞎 Cancer Guide | 🞎 Website/Other ______________________________ | |

C. Who helped you understand the side effects of chemotherapy the best? (check ONE)

| 🞎 Oncologist | 🞎 Oncology nurse | 🞎 Chemotherapy nurse |
| --- | --- | --- |
| 🞎 Chemotherapy class | 🞎 Pharmacist | 🞎 Pamphlets/documents provided |
| 🞎 Cancer Guide | 🞎 Website/Other ______________________________ | |

D. Who did you most want to help you understand the side effects of chemotherapy? (check ONE)

| 🞎 Oncologist | 🞎 Oncology nurse | 🞎 Chemotherapy nurse |
| --- | --- | --- |
| 🞎 Chemotherapy class | 🞎 Pharmacist | 🞎 Pamphlets/documents provided |
| 🞎 Cancer Guide | 🞎 Website/Other ______________________________ | |

E. When was this information provided?

| 🞎 Immediately following diagnosis | 🞎 During your oncologist visit | 🞎 Right before beginning treatment |
| --- | --- | --- |
| 🞎 Other ______________________________________________________________ | | |

F. When did you want this information provided?

| 🞎 Immediately following diagnosis | 🞎 During your oncologist visit | 🞎 Right before beginning treatment |
| --- | --- | --- |
| 🞎 Other ______________________________________________________________ | | |

1. A. Did you receive information about the financial costs of chemotherapy (i.e. reimbursement related things)?

| 🞎 Yes | 🞎 No |
| --- | --- |

B. If yes, who provided this information? (check all that apply)

| 🞎 Oncologist | 🞎 Oncology nurse | 🞎 Chemotherapy nurse |
| --- | --- | --- |
| 🞎 Chemotherapy class | 🞎 Pharmacist | 🞎 Pamphlets/documents provided |
| 🞎 Cancer Guide | 🞎 Website/Other ______________________________ | |

C. Who helped you understand the financial costs of chemotherapy the best (i.e. reimbursement related things)? (check ONE)

| 🞎 Oncologist | 🞎 Oncology nurse | 🞎 Chemotherapy nurse |
| --- | --- | --- |
| 🞎 Chemotherapy class | 🞎 Pharmacist | 🞎 Pamphlets/documents provided |
| 🞎 Cancer Guide | 🞎 Website/Other ______________________________ | |

D. Who did you most want to help you understand the financial costs of chemotherapy (i.e. reimbursement related things)? (check ONE)

| 🞎 Oncologist | 🞎 Oncology nurse | 🞎 Chemotherapy nurse |
| --- | --- | --- |
| 🞎 Chemotherapy class | 🞎 Pharmacist | 🞎 Pamphlets/documents provided |
| 🞎 Cancer Guide | 🞎 Website/Other ______________________________ | |

E. When was this information provided?

| 🞎 Immediately following diagnosis | 🞎 During your oncologist visit | 🞎 Right before beginning treatment |
| --- | --- | --- |
| 🞎 Other_______________________________________________________________ | | |

F. When did you want this information provided?

| 🞎 Immediately following diagnosis | 🞎 During your oncologist visit | 🞎 Right before beginning treatment |
| --- | --- | --- |
| 🞎 Other ______________________________________________________________ | | |

1. A. Did you receive information about the logistics of chemotherapy (i.e. scheduling and the sequencing of treatment)?

| 🞎 Yes | 🞎 No |
| --- | --- |

B. If yes, who provided this information? (check all that apply)

| 🞎 Oncologist | 🞎 Oncology nurse | 🞎 Chemotherapy nurse |
| --- | --- | --- |
| 🞎 Chemotherapy class | 🞎 Pharmacist | 🞎 Pamphlets/documents provided |
| 🞎 Cancer Guide | 🞎 Website/Other ______________________________ | |

C. Who helped you understand the logistics of chemotherapy the best (i.e. scheduling and the sequencing of treatment)? (check ONE)

| 🞎 Oncologist | 🞎 Oncology nurse | 🞎 Chemotherapy nurse |
| --- | --- | --- |
| 🞎 Chemotherapy class | 🞎 Pharmacist | 🞎 Pamphlets/documents provided |
| 🞎 Cancer Guide | 🞎 Website/Other ______________________________ | |

D. Who did you most want to help you understand the logistics of chemotherapy the best (i.e. scheduling and the sequencing of treatment)? (check ONE)

| 🞎 Oncologist | 🞎 Oncology nurse | 🞎 Chemotherapy nurse |
| --- | --- | --- |
| 🞎 Chemotherapy class | 🞎 Pharmacist | 🞎 Pamphlets/documents provided |
| 🞎 Cancer Guide | 🞎 Website/Other ______________________________ | |

E. When was this information provided?

| 🞎 Immediately following diagnosis | 🞎 During your oncologist visit | 🞎 Right before beginning treatment |
| --- | --- | --- |
| 🞎 Other ______________________________________________________________ | | |

F. When did you want this information provided?

| 🞎 Immediately following diagnosis | 🞎 During your oncologist visit | 🞎 Right before beginning treatment |
| --- | --- | --- |
| 🞎 Other ______________________________________________________________ | | |

1. Did the printed documents (i.e. pamphlets, note sheet, etc) you receive provide enough information?

| 🞎 Too little information | 🞎 Just enough information | 🞎 Too much information |
| --- | --- | --- |

1. Other comments related to your experience with chemotherapy education and the process?

_______________________________________________________________________

**Thank you for completing this questionnaire.**

**If you have any questions, please contact:**

**Scott Okuno, MD**

**Mayo Clinic Health System**

**Eau Claire-Luther Campus**

**1400 Bellinger Street P.O. Box 1510**

**Eau Claire, WI 54702-1510**

**715-838-6072**

**When you have finished this questionnaire, please hold on to it and drop it off at the Front Desk on your way out.**

**12/19/2018**

Used with permission of Mayo Foundation for Medical Education and Research.
